# Using intersectionality to explore physical activity participation amongst individuals with vision loss from ethnic minority backgrounds in the United Kingdom

**DOI:** 10.1038/s41598-026-55909-8

**Published:** 2026-06-02

**Authors:** Peter M. Allen, Deanna Finn, Paul Tracey, Thomas E. D. Hall, Shabana Younus

**Affiliations:** 1 Vision and Hearing Sciences Research Centre, Anglia Ruskin University, Cambridge, CB1 1PT UK; 2 Department of Psychology, Anglia Ruskin University, Cambridge, UK; 3 Judge Business School, University of Cambridge, Cambridge, CB2 1AG UK

**Keywords:** Health policy, Public health

## Abstract

Participation in physical activity (PA) is essential for maintaining health and mobility. Although women, individuals from ethnic minority backgrounds, and people experiencing vision loss are reportedly the least active of their respective identity classifications literature is yet to acknowledge the intersectional nature of group identification, and the ways in which barriers and facilitators to PA might vary or compound in instances of multiple-minority status. Fifteen people with vision loss (six male) from different cultural backgrounds (six White: nine South Asian) were interviewed. Reflexive thematic analysis, underpinned by an interpretivist epistemology and relativist ontology, was used to analyse data. Participants identified neglect at a governmental level as the most significant barrier to PA, producing inaccessible environments that are infrastructurally inequitable. Female South Asian participants’ barriers to PA were nuanced by the intersectionality of their multiple identity positioning (gender, race, and disability). The cultural expectations placed on South Asian women, specifically those pertaining to modesty and gender, limited their capacity to engage in a physically active lifestyle. Comparisons made between the experiences of White and South Asian participants highlighted a lack of acceptance towards disabled individuals in the South Asian community. This study provided insight as to how experiences of inequality intersect and require holistic means of intervention. Governmental bodies and social innovators should strive to create spaces which are architecturally appropriate for people with vision loss, and work with sporting facilities such as gyms to centralise marginalised communities in their design to provide services which are both accessible and culturally viable.

## Introduction

Globally it is estimated that there are over 2.2 billion people living with vision loss which has a significant impact on their daily lives^1^. In the UK this equates to approximately 2 million people living with vision loss. By 2050 the number of people living with vision loss in the UK is predicted to increase to over 4 million^2^. Having a vision impairment can impact all aspects of a person’s life and has been consistently linked to poorer physical^3^ and mental^4^ health. Thus, interventions which reduce the risk of poor physical health are essential for tackling health inequalities and lower life expectancy among populations with vision loss. This research is motivated by a desire to inform the design of programs that facilitate greater uptake of physical activity (PA) among South Asian women with vision loss, a disproportionately disenfranchised population. Social innovation refers to the design and implementation of holistic interventions that serve to incite changes in social policy^5^. Specifically, social innovation allows for the development of policy and rehabilitative programmes which acknowledge the multiple and interlocking sources of influence that may prevent an individual or certain population(s) from accessing the resources they require^6^.

Intersectionality^7^ examines how interlocking systems of power manifest in individual experiences. Intersectionality explores how one’s social positioning is dependent on affiliation within differing minority classifications, and how multiple identity labels have the potential to offer unique opportunities for privilege and sources of oppression^8,9^. As a result of the overlap of differing inequalities, an individual’s experiences of barriers to and facilitators of PA often manifest at varying intersections of sex/gender, race/ethnicity, and disability^10^. Using an intersectional lens allows for contextualisation of the unique and variable barriers faced by minority populations and the complex interplay of multiple identities^8^.

In the context of this study, PA will be defined as ‘any bodily movement produced by skeletal muscles which requires energy expenditure, this includes activities undertaken whilst working, playing, carrying out household chores, travelling, and engaging in recreational pursuits.’^11^. In 2018, 44% of adults did not meet the recommended 150 min of moderate PA or 75 min of vigorous PA per week, and 22% of adults did less than 30 min of PA per week^12^. Low levels of PA is reported to contribute to one in six deaths in the UK, and evidence suggests PA levels are continuing to decline, with estimates predicting the population will be 35% less active by 2030 than populations were in the 1960s. Disabled populations, women, and South Asian communities, are frequently reported as being the most inactive of their respective identity classifications. Diagnosis of vision impairment is generally associated with additional health implications, and an overall decline in general wellbeing^13–15^. These comorbidities can largely be attributed to an increase of sedentary behaviour post-diagnosis as people with un-correctable sight loss are twice as likely to be inactive than those without sight loss^16^. Furthermore, it has been argued that those with disabilities engage in less physical activity than their able-bodied peers because they have fewer opportunities to do so, and in some cases are disabled more by their environment than their medical condition^17^. Inaccessibility at an architectural level is a significant barrier to physical activity for those with mobility, hearing, and visual disabilities^18,19^. Navigation around inaccessible environments can be stressful and anxiety inducing for those with sight loss as they are largely unpredictable and difficult to account for^19^. In terms of insensitivity, disabled populations report that the needs of mobility and vision impaired individuals are rarely acknowledged in the design of public spaces. For example, the current modernisation of gyms whereby buttons are replaced by touch-screen interfaces is concerning as it is frequently becoming the case that gym equipment lacks the tactile and haptic feedback necessary to be operated by individuals with sight-loss^20^.

Likewise, women are disempowered from PA participation by the gendered roles enforced by wider society as they are taught that they must excel as mothers, wives, students, and employees^21,22^. This can create role conflict whereby women struggle to manage all the identities they are expected to conform to^21^. Women have been found to engage in more unstructured, home-based exercises than men. Coen et al.^23^ suggest that this reflects an attempt on behalf of women to make time for physical activity in a constrained environment whilst juggling the general domestic demands of family life. Men face less resistance in engaging in sporting behaviours as they have more flexibility and recreational freedom as their mothers, sisters, wives, and daughters manage household responsibilities and childcare^23^. Women are systemically barred from engaging in recreational pursuits as physical activity is framed as being an additional burden to make time for alongside familial and domestic obligations^22^.

Finally, despite increased awareness of the links between physical inactivity and debilitating long-term chronic illness, it is consistently found that those from minority ethnic backgrounds represent a small proportion of those who meet governmentally advised guidelines around maintaining a physically active lifestyle^24,25^. Whether it be recreational, domestic, or occupational, racial minorities are consistently found to engage in more sedentary behaviour than dominantly situated populations^24^. South Asian girls are typically taught to maintain the household as well as clean up and care for their male siblings to prepare them for marriage where they are traditionally expected to sacrifice their independence and social freedom for the sake of supporting their family^26^. Women who prioritise their health are at greater risk of being ostracised by the local religious community as recreation and leisure-based pursuits are often stigmatised and deemed a distraction from domestic obligations, as well as associated with the neglect of familial responsibilities^26,27^. These communities also place a great emphasis on modesty, with South Asian women referencing gym clothing, advertisements, security cameras, the presence of male staff, and mixed gender facilities as some of the major deterrents from engaging in organised physical activity^28^. Whilst each of these identities (disabled, South Asian, and woman) have been investigated in relation to the uptake of PA to a certain degree, there is a need to investigate the intersectionality of all three combined.

In an interesting study, Burton et al.^29^ suggests that facilitators and barriers to PA for people with vision loss are experienced in three ways: (1) psychologically; (2) through opportunity and access; (3) and at a societal and policy level. These three influences can be considered in relation to Crawford et al.’s^30^‘hierarchical model of leisure constraints’ which proposes that barriers to PA are found at three levels: intrapersonal, interpersonal, and structural (e.g., availability of PA, environments, and resources). Crawford et al.^30^ suggest these barriers are hierarchical with intrapersonal constraints required to be overcome before interpersonal and structural ones.

Lee et al.^31^ states that “the use of population-based, large-scale surveys could better inform more inclusive PA promotion policies and programs”. Though while population-based surveys are useful, they often miss the subtle nuances that can only be elucidated by focussing on individuals within a local setting. Moreover, Lehne and Bolte^32^ suggest that there is an urgent need for transparent reporting of social inequality-related findings related to programs designed to engage people in PA as there is an accumulation of evidence suggesting that universal interventions to improve levels of PA are successful at a population level, however they may widen social inequalities between different ethnic groups. This has been termed “intervention-generated inequality”^33^. Therefore, using an intersectional lens may help illuminate why PA participation in marginalised groups continues to be very low when compared to their counterparts who are dominantly situated^8^. As such, the present study will utilise qualitative interview data to compare the experiences of six White and nine South Asian male and female participants with un-correctable vision loss to gauge whether the barriers to and facilitators of PA noted by these populations compound in the instance of multiple-minority identification.

This paper explores the following research question:

- How do intersecting identities (race, gender, and disability) shape the barriers to and facilitators of physical activity for individuals with vision loss?

## Methods

### Participants

Purposive sampling was employed to recruit participants through a variety of vision loss charities (via their social media channels), religious organisations, and eye clinics. Recruitment proved challenging with a limited uptake from advertisements, participants were selected if their demographic profile aligned with the issues central to the research goals^34^, primarily if they were affected by significant vision loss, allowing for a thorough appraisal of potential contributors to the lack of uptake of PA in specific populations. Fifteen individuals with vision loss (F:9, M:6) participated in the study, demographic information is summarised in Table 1. Participants were divided into 5 groups based on gender and ethnicity to allow for differentiation between issues specific to vision loss, gender, and ethnicity/religion/culture. Groups were as follows: White male, White female, Muslim Asian female, Muslim Asian male, additionally three non-Muslim Asian females were recruited to assess the significance of faith.

**Table 1:** Participant characteristics.

Gender	Age	Ethnicity	Religion	Impairment
Female	48	White British	Christian	VI
	68	White British	Christian	SVI
	48	White Romanian	Christian	SVI
	58	British Pakistani	Muslim	SVI
	54	British Pakistani	Muslim	SVI
	51	British Pakistani	Muslim	SVI
	52	British Indian	Hindu	SVI
	70	British Indian	Hindu	SVI
	64	British Indian	Christian	VI
Average age	(57)			
Male	34	White British	Christian	SVI
	47	White British	Christian	SVI
	47	White British	Christian	SVI
	55	British Pakistani	Muslim	SVI
	62	British Pakistani	Muslim	SVI
	39	British Pakistani	Muslim	SVI
Average age	(47.3)			

Note. The mean age of all participants was 53.1 ± 10.1 years. VI = vision impaired, SVI = severely vision impaired (blind).

### Data collection

Prospective participants were contacted via email and/or telephone where they were given a brief overview of the study and invited to participate. People who showed interest were contacted and forwarded a survey. Once completed and returned, a video interview was arranged. All participants gave informed consent twice, once at the start of the survey, and a second time before the video interview was conducted. All participants had capacity to give informed consent. Ethical approval for the study was obtained through the University of Cambridge, Cambridge Judge Business School’s standard process for student research. All methods were performed in accordance with relevant guidelines and regulations.

The interview script covered the following sections: background information about the participant, understanding and level of PA involvement currently, facilitators and barriers to doing PA, and wider influences on participation in PA. Each section consisted of questions designed to produce information about the participants’ experience of PA participation. Verbal probes such as “Can you explain a bit more?” or “How did that make you feel?” were used to elicit more information. The interview template is included below (Table 2):

**Table 2:** Interview questions/template.

Category	Example questions
Background	Tell me more about yourself (family, work, religion etc.) Would you mind telling me about your vision loss (visually impaired or severely visually impaired? ).
Physical activity	I wanted to ask more about your physical activity. Firstly, what would you understand by the term physical activity? How do you feel about physical activity in general? What physical activity do you do (prompt if needed as not just sport)? How often are you doing these? How easy is it to do these activities? Why? Is physical activity important to you? Expand.
Facilitators	What do you enjoy about doing your physical exercise activities? What don’t you enjoy about them? What do your family/ friends think about your physical activity? What would encourage you to do more physical activity?
Barriers	What makes it hard to do physical activity? Any other challenges or obstacles – transport? Cost? Gender? Religion? Culture? Ethnicity? Vision loss? Do you feel you can decide how much physical activity you do, or is that not in your control? Why? What would help you fit in more physical activity? Anything else that has helped?
Wider influences	What physical activity do your family/ friends do? Are there any other factors that affect what you do regarding physical activity that you have not mentioned? (If not mentioned above). What do you think about doing physical activity with other people? – mixed gender? Mixed vision loss? Where would be the best place for you to do physical activity?

The interview process was first pilot tested on three women with vision loss (not included in the study). Following this, amendments were made to the wording and order of questions to ensure that participants fully understood the questions being asked, that the questions were addressing the specific aims of the study, and that the flow of the interview was logical. Semi-structured interviews were conducted online via Microsoft Teams and recorded (and transcribed) using the respective software following the permission of the participant. The interviews were conducted from December 2023 to March 2024. The authors determined that data saturation was reached, and no further interviews were required.

### Positioning and researchers

This research is positioned within an interpretivist epistemology and relativist ontology. Specifically, it was recognized that knowledge is not a matter of discovering objective facts about the world, rather, it is created through the interaction of individuals and their social context. By emphasizing the subjective and social construction of reality it is emphasised that the meaning of the event in question (the experience of participating in PA for women from ethnic minorities with vision loss) is constantly negotiated and reinterpreted by the people involved^35^. Thus, interpretivism is particularly suitable when aiming to understand the meaning of people’s experiences in detail, and from their own perspective. Relativism holds that no single, objective reality exists, instead there are multiple realities that are constructed by individuals and groups through their own experiences and perspectives, each of which are valid^36^, thus this position is coherent with an interpretivist epistemology.

The lead author (PMA) is a white British male with an academic professorship in optometry. PMA’s professional experience of individuals with vision loss was seen by all authors as a strength of this research as he was sensitised to the nuances of vision loss and its effects in practice. Furthermore, throughout this research a conscious effort was made to minimise confirmation bias, the tendency to elicit information that supports your views or perceptions^37^ as it was acknowledged that there is a tendency for researchers to identify confirming evidence, and significantly more difficult for researchers to identify or evaluate evidence that is contrary to their expectations^38^. Consequently, the fifth author (SY), a female Muslim conducted the interviews due to the argument that the greater the racial/ethnic similarity between the researcher and the researched the greater the likelihood of disclosure and establishing a more egalitarian research relationship^39^. Papadopoulos and Lees^40^ advocate a strategy of ‘ethnic matching’ between researchers and research participants. PMA and SY regularly discussed and reflexively challenged one another’s assumptions about the participants’ experiences.

### Data analysis

Reflexive thematic analysis was used to analyse the data and identify meaningful patterns, with two researchers conducting independent analyses before collaboratively generating results. Such an approach is appropriate given the interpretivist positioning. The transcripts were checked by the two researchers who were present at the interview (one actual interviewer and the second who was always in the background) for transcribing errors within 2-days of the interview taking place. Any changes were checked and confirmed (or rejected) by unanimous decision of both researchers.

The method suggested by Braun and Clarke^41^ was used to direct the thematic analysis. This included (1) immersion and familiarisation of the data by reading and re-reading the interview transcripts and making notes on any initial observations made, (2) further reading of the data set to generate initial codes. The identified concepts were useful to create first-order codes following an inductive analysis process. Subsequently the codes were expanded, refined, renamed, and more interpretive codes developed, (3) the first-order codes were axially coded by moving reciprocally between the interview data, the literature and the first-order codes. This enabled grouping of first-order codes into second-order overarching identities (for both research questions). Consequently, the codes were organised into groups that were deemed as potential themes based on their shared, implicit, and explicit concepts, (4) the themes and codes were reanalysed using an iterative process^42^ to ensure coherence and relevance to the research questions and (5) the theme names and their definitions were finalised. Methodological quality was judged against the twenty critical questions for quality reflexive thematic analysis coined by Braun and Clarke^43^.

## Results

Participants were asked a preliminary question to provide an explanation of what ‘physical activity’ meant to them. Responses varied, some believed PA to refer exclusively to moderate-vigorous cardiovascular exercise, e.g., ‘*Something that gets your heart rate up.’ [Female (48)*,* White British*,* Christian].* While others spoke in a broader sense, suggesting that PA might encompass daily chores or domestic duties; ‘*Doing the dishes for me at least could be exercise.’ [Female (51)*,* British Pakistani*,* Muslim]*. Generally, participants conceptualised PA as ‘*Just keeping my body active.’ [Female (70)*,* British Indian*,* Hindu]*. The following analysis will utilise the latter definition.

**Fig. 1:**
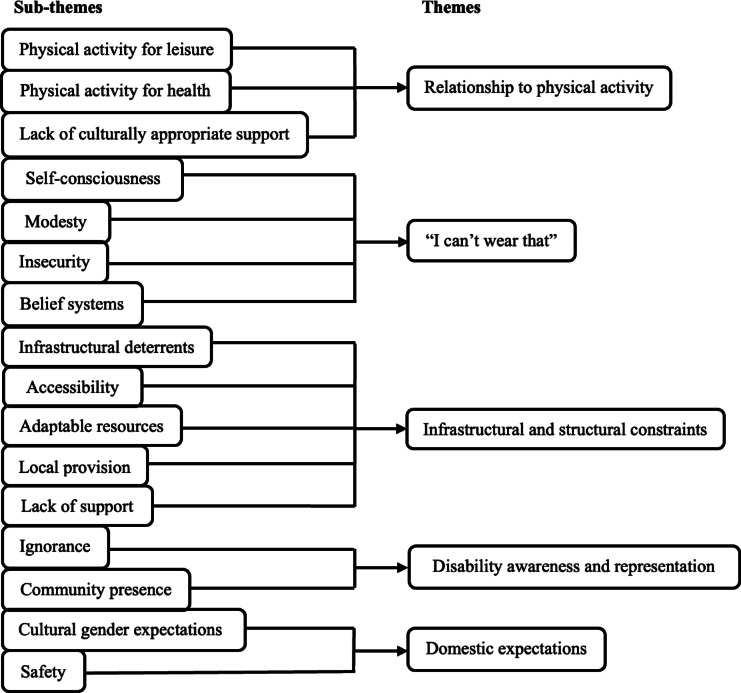
Thematic map. Note. A thematic map displaying the sub-themes (left) that were used to construct themes (right).

Five themes were generated through thematic analysis: relationship to physical activity, “I can’t wear that”, infrastructural and structural constraints, disability awareness and representation, and domestic expectations. Themes were constructed out of sub-themes that were prominent throughout transcripts (see Fig. 1). Within these themes, the intersecting nature of disability, gender, and ethnicity is discussed.

### Relationship to physical activity

Participants were asked to describe their relationship with PA and current activity levels. Male participants appeared to engage in more PA than female participants, offering numerous and varied examples of moderate to vigorous sporting activities: *‘I’ve always tried to keep myself reasonably fit. I do play disabled football and I’m trying to sort of look around for other sports.’ [Male (47)*,* White British*,* Christian].* While Female participants admitted to generally finding PA a struggle. They emphasise their vision loss as being a significant barrier to participating in any form of bodily movement: *‘I’m registered totally blind. I can’t see my feet*,* so basically*,* I’m unable to walk up and down the street’ [Female (51)*,* British Pakistani*,* Muslim].*

This was nuanced further, with data evidencing a significant racial/cultural divide in the way that individuals detailed their involvement in PA. Dominantly situated participants began by describing their engagement with PA as a means of recreation or leisure, deriving enjoyment and a sense of fulfilment from bodily movement: *‘I like the way exercise makes me feel. I enjoy the socialisation of physical activity.’ [Female (64)*,* White British*,* Christian]*. While participants from a minority ethnic background often referred to PA in a more prescriptive sense, highlighting its role both as a means of prevention as well as recovery from long-term chronic illness as key motivators for their participation: *‘To improve my cholesterol. My motivation is to lose weight because I’m diabetic.’ [Female (58)*,* British Pakistani*,* Muslim]*, additionally: *‘To prevent depression*,* for my mental health it’s important.’ [Male (55)*,* British Pakistani*,* Muslim].*

When directly asked if they believed that their race/ethnicity played a unique role in the barriers they faced, participants often disagreed, though would later go on to suggest otherwise. For example, one participant mentioned that the support offered to individuals with vision loss, such as using a guide dog, is not always appropriate as it conflicts with their religious practice: *‘I don’t like dogs and secondly it wouldn’t do because of my prayer and all that*,* it breaks our prayer and wudu.’ [Female (51)*,* British Pakistani*,* Muslim]*. For this participant, specific religious ritual requirements rather than race/ethnicity alone are preventing her from accessing the mobility benefits offered by guide dogs and subsequently from accessing PA.

### “I can’t wear that”

This theme encompasses those intrapersonal and psychological factors which may hinder the uptake of a new habit. All participants were able to evidence a reasonable understanding of the health benefits associated with continuous involvement with PA, demonstrating that they possessed both the drive and motivation to adopt a more active lifestyle. However, psychological constraints and insecurity were prevalent throughout the transcripts. A commonality amongst participants was their general reliance on the assistance of others (namely family and friends), but some expressed concerns that they were ‘*always a burden to other people.’ [Male (62)*,* British Pakistani*,* Muslim].* Others displayed a heightened concern over how they are perceived by others, *‘You don’t know where you’re going to touch a person*,* all that sort of stuff because you don’t know where they are*,* and so that sometimes does play a role. So*,* I avoid going into swimming pools where there’s a lot of people’ [Male (55)*,* British Pakistani*,* Muslim].*

When internalised, these beliefs can act as major psychological barriers to engaging in PA and prevent individuals from receiving the support they need. One participant highlighted how social beliefs regarding the capabilities of blind people significantly affect one’s outlook post-diagnosis. A re-framing of cultural beliefs to acknowledge what one is capable of rather than emphasising their impairment can foster optimism and greater willingness to explore physically challenging lifestyles. *‘Some of the biggest barriers are social and like a person’s own belief system*,* like in terms of what they believe they can achieve*,* what they can do. If you say to someone they can’t see properly*,* they’re blind straight away. A part of them switches off and they believe that they are no longer of any use and can no longer do anything’ [Female (68)*,* White British*,* Christian].*

Whilst these experiences were shared more universally amongst participants due to being comparatively visually impaired, the South Asian female participants’ experience was modulated further by the modesty expectations of their respective religions. They described their struggle in maintaining modesty whilst engaging in PA as they are, ‘*fully covered with baggy clothes and a head scarf.’ [Female (54)*,* British Pakistani*,* Muslim].* Some worry for how they are being perceived, as exampled in: *‘I have to wear clothes that other people don’t have to wear.’ [Female (70)*,* British Indian*,* Hindu].*

One participant expressed concern over being asked to refrain from wearing headscarves whilst exercising. Lack of awareness or appropriate sensitivity training results in some minority populations being unnecessarily excluded from sporting activities. *‘I think they don’t recommend because of health and safety. You know when you train*,* most of your body heat comes from your head. I’m not sure if there’s any difference to people wearing caps. Like people wear caps and your head is still covered*,* and it all gets quite sweaty under there as well.’ [Female (51)*,* British Pakistani*,* Muslim]*

While some participants describe the outer-wear of other female gym-goers as significant deterrents to attending public facilities and spaces dedicated to sport. *‘Like with gymnastics for example*,* you do have to wear certain outfits. I prefer dance because you can wear normal clothes. Some women wear very tight clothing. I can’t wear that*,* and I wouldn’t feel comfortable if that is what is expected to wear.’ [Female (70)*,* British Indian*,* Hindu]*

Overall fear of ridicule, discrimination, and social exclusion were evident throughout these narrative accounts. It is clear from these results that the South Asian women in this study face more barriers than any other minority population due to heavy cultural restrictions regarding how they must engage with PA, they face pressure to respect traditional Islamic practices by exhibiting modesty and maintaining their femininity or otherwise risk social ridicule as well as having their piety and religious devotion brought into question.

### Infrastructural and structural constraints

This theme mentions external influences that are largely beyond individual control, being instead the responsibility of local councils/government organisations, but also within sports and fitness facilities themselves. Participants described their struggle in finding local opportunities to engage in PA and arrange travel: *‘I struggle to participate and find opportunities that are accessible. I normally have to travel further afield. The council gym has recently shut down in the town I live.’ [Male (39)*,* British Pakistani*,* Muslim].* Dissatisfied with local provision of PA opportunities, one participant eloquently described the issue: *‘The responsibility with fitness and all this health should lie with the local authority or government. The problem is*,* once you put all this in a private setting*,* there is only one winner – that’s the people who can afford to use these facilities*,* which excludes a hell of a lot of people*,* especially people who are disabled and people who cannot live independently.’ [Male (62)*,* British Pakistani*,* Muslim].*

For those who did have more local opportunities, active means of travel (such as walking) were said to incite anxiety. Raising awareness of the struggles faced by this population may facilitate greater engagement in outdoor PA as negligence (taking the form of unpredictable obstacles) was noted as being the root cause of these anxieties, although these often took many forms. For example, one participant mentioned, *‘It’s how people park their cars and leave the dustbins outside.’ [Female (70)*,* British Indian*,* Hindu]*, while another claimed that their confidence in navigating their environment and travelling independently had only decreased in recent years with the advent and popularisation of electric motors: *‘Electric cars are very good for the environment and all that stuff*,* but they’re very bad for us. We can’t actually hear them*,* so when you’re crossing the road*,* you have to be extra careful. We rely on our ears.’ [Male (55)*,* British Pakistani*,* Muslim]*

An architecturally hostile environment poses a significant risk to the disabled persons’ health and safety leaving them dependent on means of transportation that may be unreliable and inconvenient. This effectively deters disabled populations from engaging in PA in the first instance: *‘I spend more time travelling there than actually doing any exercise. It’s not really worth it.’ [Male (39)*,* British Pakistani*,* Muslim]*. The absence of appropriate accommodation for PA participation restricts this population’s autonomy and sense of independence.

Additionally, sporting facilities were often deemed inaccessible, restricting visually impaired gym-goers in terms of what they can access, when, and where. It was almost universally agreed that *‘Gyms are not designed for people that are blind.’ [Female (51)*,* British Pakistani*,* Muslim].* Obstacles such as sporting facilities being ‘*really busy [with] loud music playing and people all over the place and it’s quite difficult to orient yourself.’ [Male (39)*,* British Pakistani*,* Muslim]*, affected participants at all levels of fitness. Gym equipment was highlighted as being a significant issue, the lack of tactile and haptic feedback was said to be dangerous and made some feel as though their *‘independence was being taken away by some pieces of equipment.’ [Female (64)*,* White British*,* Christian].* For example, one participant stated: *‘They could make the machines more accessible*,* like putting tactile markings on them for different weights or settings. A lot of the machines are touchscreen now*,* so they are not easy to use.’ [Male (39)*,* British Pakistani*,* Muslim]*

Multiple participants expressed concern over the lack of support provided by gym staff. Some found the company of a friend or partner reassuring, increasing their confidence in navigating facilities and utilising equipment. Participants expressed a desire to see these feelings of support reflected in their interactions with staff, suggesting that the presence of a guide would be beneficial and make sporting environments less intimidating. As it currently stands, participants described staff as being inexperienced and untrained: *‘I’m looking around like an idiot*,* and I think*,* you know*,* you’re not trained. It puts you off so this it just kind of ruins your whole motivation for going out.’ [Female (51)*,* British Pakistani*,* Muslim]*

This is a demonstration of the social relational model of disability^44^ whereby disability is being imposed on a population by society, with inaccessible facilities, lack of support, and hostile architecture limiting PA opportunities, rather than the visual impairment itself. Further emphasising this point in a more positive way, participants highlighted ways in which adaptable behavioural intervention programmes and gyms were able to facilitate greater participation in PA through the provision of adaptable resources which could accommodate their specific needs: *‘Things like adapting the game*,* so using audio more. That just makes you feel a bit safer when you’re out playing’ [Male (47)*,* White British*,* Christian].*

Additionally, participants mentioned how they were able to maintain active lifestyles throughout COVID due to online resources which prioritised communication and inclusivity: *‘During lockdown I was doing online yoga classes*,* and the teachers were really good because they would just describe in great detail how to do each move. If I was doing it wrong*,* they would say “Oh yeah*,* you’re doing it this way*,* you have to do it like this”.’ [Male (39)*,* British Pakistani*,* Muslim].*

### Disability awareness and representation

This theme collects participants’ reflections on their experiences of ignorance towards disability and lack of representation in PA settings. These concepts seemed especially prevalent amongst the South Asian participants, suggesting a strong influence of ethnicity over those factors limiting PA involvement.

One participant claimed that disability awareness is not as widespread in this population, stating that members of the Asian community ‘*feel that there isn’t a need for that’* (‘that’ meaning intervention or established systems of support) *[Male (39)*,* British Pakistani*,* Muslim].* Limited knowledge and understanding became a prevalent sub-theme, participants stressed an unspoken rule that disability was not discussed within the South Asian community, and physically active lifestyles were rarely modelled: *‘Cultural things like my parents have never done physical exercise. A lot of the time they expect you to stay at home’ [Male (39)*,* British Pakistani*,* Muslim]. ‘I think it was because they just didn’t have the knowledge and understanding*,* and to them anything that they don’t understand is best not spoken about. I think they like to brush it under the carpet and put it out of sight.’ [Male (39)*,* British Pakistani*,* Muslim].*

To add to this point on culture, one participant said, *‘There is a cultural issue here as well*,* the Asian culture. Sometimes you find that as visually impaired*,* [you] find it difficult to ask people for help’ [Male (55)*,* British Pakistani*,* Muslim].* Resulting interactions with able-bodied members of the Asian community lack sensitivity, for example, when asking for help, some participants mentioned that: *‘The first thing that comes back is not “Yes I’ll help you cross the road”*,* the first thing they say is “What are you doing outside of the house?”’ [Male (55)*,* British Pakistani*,* Muslim].*

In PA settings, South Asian participants often mentioned the lack of representation in gym staff, volunteering roles, and in other supporting capacities as a major deterrent for participating in PA and group sporting events. Some felt that systems of support should reflect the community it serves, suggesting that identification was important for fostering a sense of community and facilitating greater acceptance of help: *‘In terms of volunteering and kind of helping with visually impaired people*,* there’s not many Asian people that do that.’ [Male (55)*,* British Pakistani*,* Muslim]*. When asked why this was the case, it was suggested that the culture of ambition, hyper-independence, and the emphasis on attaining a respectable career and financial security led some to ignore more community-focused means of inciting change: *‘We’re just kind of career driven. I think that they’re more focused on work. Volunteering is not something that they think about.’ [Male (39)*,* British Pakistani*,* Muslim]*.

### Domestic expectations

This theme explores the notion arising from the interview data that, generally, there is a cultural expectation on South Asian females to ‘*not go out of the house*,*’* and instead engage in less vigorous PA that is domestic in nature, and beneficial for the household. When asked how these expectations change or differ in the context of female disability, one participant claimed they were just as stringently enforced: *‘Yesterday I did the cooking for the family as normal.’ [Female (52)*,* British Indian*,* Hindu].* These gendered roles are placed on women regardless of their capacity to fulfil them. The same participant went on to highlight the disparity between disabled men and women, implying that disabled South Asian females feel compelled to engage in PA that they do not necessarily derive any pleasure from: *‘Females [are] required to be more active as they are still expected to fulfil domestic obligations. When the male probably has an issue like this*,* they don’t do much because their wife does it for them.’ [Female (52)*,* British Indian*,* Hindu].*

A male participant echoed this sentiment, though suggested that safety was also a pertinent issue. Both participants statements refer to Asian women with vision loss as though decisions around their participation in PA are made on their behalf and largely beyond their control. *‘It would be even harder if you were a female of a Pakistani background. Women are expected to not go out of the house especially*,* like if you’ve got a vision impairment*,* [parents] will be more protective and say like “oh something might happen to you” or “you might get attacked.”’ [Male (39)*,* British Pakistani*,* Muslim].*

## Discussion

Through conducting interviews with men and women with vision loss of varied ethnic/race identities, this study explored whether the barriers to PA experienced by disabled individuals (people with a vision impairment), women, and ethnic minorities would be nuanced by multiple minority identification. Analyses benefitted from utilising an intersectional framework. The barriers and facilitators to engagement with PA will now be discussed, firstly by focussing on women with vision loss from ethnic minority groups, before discussing more broadly individuals with vision loss.

### Barriers to and facilitators of PA involvement amongst women with vision loss from ethnic minority groups

In contrast to current literature on the subject, this study would suggest that women from the South Asian visually impaired community engage in more PA overall than men as they have no choice but to do so. In South Asian communities, women are generally raised to believe that they have an inherent proclivity towards domestication, that they must centre their lives around hospitality and familial commitments, and that pursuing goals outside of their household is selfish and goes against their nature^21,22,45^. Women are expected to manage most if not all domestic labour, prioritising the provision of food, clothing, and shelter for their children at the expense of their own personal wellbeing and physical fitness^46,47^. Both male and female South Asian participants implied that decisions regarding a women’s participation in PA were made on their behalf, the cultural expectation that women must be homemakers leaves other members of a household dependent on female inhabitants regardless of their capacity to fulfil such obligations. Whereas ethnic minority males with vision loss were permitted more opportunities for rest as they are taught to rely on the women around them. These assertions imply that disabled Asian women feel compelled to engage in domestic-based PA that they do not necessarily derive any pleasure from. Therefore, the cultural expectations of South Asian women act as a facilitator of PA, but only of that PA which is domestic and obligated, whereas these same cultural expectations block their participation in recreational PA or PA for leisure. The findings of this study support the work of Peng and colleagues^22^, who have previously noted that Muslim and other South Asian women are not as exercise averse as wider literature suggests, but rather these women must adhere to a hierarchy where cultural norms and religious practices are prioritised, taking precedence over self-serving individual attempts at improving physical health. The intersectionality of feminine gendered expectations combined with the restrictions enforced through cultural and religious practices create a unique barrier to recreational PA, but a facilitator of overall PA, experienced exclusively by South Asian women. While the South Asian women in this study spoke of PA in a purely low-intensity, and domestic sense (exampling means of bodily movement that lie beyond the typical vigorous and recreational sporting contexts), our findings offer new insight into the activity habits of a population that is often perceived as having limited engagement with physical activity.

### The influence of ethnic minority grouping on barriers to and facilitators of PA for individuals with vision loss

Participants would disagree when asked whether their racial/ethnic identity plays a role in the barriers to PA they experience, though would later go on to describe aspects of South Asian culture and social norms that are not conducive to regular engagement with a physically active lifestyle. A study by Imran et al.^48^ investigated how cultural practice influences the understanding of diabetes and the uptake of preventive self-care behaviours in South Asian populations. They found that although participants could evidence a decent understanding of the importance of engaging in self-care behaviours to maintain and regulate diabetic symptoms, this rarely translated into successful lifestyle changes that prioritised health and wellbeing^48^. The authors go on to develop the “Society-Community-Family” model of influence to demonstrate how one’s decision to engage in self-care behaviours is influenced externally by society, their local community, and family. One’s motivation to practice self-care is then dependent on several individual factors, such as their knowledge of the importance of self-care, the extent to which self-care is culturally viable, their personal capacity to incorporate and maintain appropriate lifestyle changes, and the extent to which they feel they can rely on others for support^48^.

The present study has shown how the PA habits of visually impaired South Asian populations are similarly dependent on the influence of external factors, in this case, factors such as architectural and infrastructural barriers. Inaccessible architecture for example influences one’s mobility (a personal constraint) as well as their capacity to engage in means of PA which are culturally viable by limiting opportunity and restricting access. Additionally, aspects of community enforced cultural influences could extend to the ideas of modesty and familial reputation mentioned by participants in this study. Culturally, there appeared to be a significant disparity in the perception of PA between White and South Asian participants. Dominantly situated participants spoke of PA in a recreational sense, whilst South Asian participants referred to PA as a means to an end, either fulfilling a purely medicinal role of relieving chronic pain or as part of domestic duties. These findings are consistent with previous literature, their views reflect how one’s perception of (and therefore, motivation to engage with) PA is influenced by infrastructural inequity and lack of role models or community support. Sher and Wu^49^ suggested that differences in PA between racial classifications were not due to cultural restraints or variations in habitual behavioural routines, but rather the experience of discrimination and systemic racism, with infrastructural inequity being the root cause of inactivity. Volitional and recreational exercise is one of many privileges of rich educated White males as they are more likely to have flexible work arrangements, secure accommodation, and more recreational freedom as their mothers, sisters, wives, and daughters are responsible for childcare and housekeeping^49,50^ leaving minority ethnic populations relegated to exercising within the confines of occupational and domestic obligation. These findings could be further explained by the Inverse Equity hypothesis^51^ which suggests that when new health interventions are introduced, they are embraced first by the most privileged members of society^52–54^. This leads to an initial increase of inequity, widening the gap between normative and minority populations as these interventions are rarely designed to accommodate for those of disabled, ethnically diverse, or gender minority in the first instance^51^. Our results offer support for these previous findings as White participants, despite having a visual impairment, were consistently found to regard engaging in recreational PA as a relative norm. In this sense, racial identity takes precedence over disability in the experience of privilege/oppression. Furthermore, with interventions and their associated architectural spaces favouring the racially privileged, those from minority ethnic/cultural groups have less access to safely navigable and comfortable environments within which to pursue recreational PA. Finally, our results show that motivation is a primary influence of engagement with self-care behaviour as one’s motivation to participate in PA is influenced by their capacity to do so amidst personal and societal constraints.

This research expanded current literature by providing a detailed narrative account on intersectionality of disability, gender, and race in the context of uptake of PA. This study provided an in-depth analysis on the barriers to and facilitators of participation in PA faced by women from ethnic minorities with vision loss, a group largely deemed illusive due to the compounding nature of cultural expectation, disabled inequality, and gender inequality. These results demonstrate that a Socio-Ecological Model is necessary in order to exact a holistic intervention that addresses the multiple, intersectional barriers experienced by this population. The Socio-Ecological Model is an integrated framework which acknowledges that individual development is reliant on multiple reciprocal interactions between individuals and their surrounding environment. These interactions have the opportunity to both support (facilitate) and deter (a barrier) the acquisition of new habits or behavioural routines^56^. Identifying the ways in which context influences one’s behaviour is integral for implementing behavioural interventions that are complementary to an individual’s specific situation^55^.

Additionally, future social ventures should acknowledge the barriers and facilitators experienced by disabled populations. This study’s resulting narrative accounts generally support extant literature. Participants spoke of their struggle with finding local means of engaging in PA which were both accessible and safe. Infrastructural barriers such as hostile architecture, inaccessible facilities, organisational negligence, and general insensitivity on behalf of sport providers featured prominently and were referenced as being major deterrents to engaging in PA^18,19,56^. These themes were echoed across all participants regardless of gender or racial category, highlighting environmental barriers as being a unifying experience across all disabled people. Sporting facilities, local authorities, and governmental bodies should be held accountable for this oversight by ensuring that accessibility is a priority in design. Those with disabilities will continue to be marginalised so long as they remain a peripheral concern to governmental and organisational bodies. The reliance these populations have for infrastructural support restricts their capacity for PA. They must resign to being active on other’s terms, resulting in inconsistent and fragmented patterns of PA to the detriment of their general health and wellbeing.

PA ‘prescribed’ by a health professional could be an important component of a multi-disciplinary approach to increase PA and improve the health and well-being of women with vision loss from ethnic minorities^57^. There is a plethora of terms given to interventions which include PA prescribed or promoted by a health professional, however, the uniting feature of these interventions is that a health professional gives PA advice and information to a patient with the aim of increasing the patient’s PA levels.

There were three primary limitations to this research. Firstly, barriers to PA participation were more salient than facilitators in the interview transcripts. This resulted in a limited discussion of facilitators to PA in the subsequent analysis. It may be that for this population, the barriers do outweigh the facilitators, but on reflection, interview questions could have more thoroughly probed for facilitating experiences of PA to the same extent as the barriers. Secondly, recruitment was challenging, and participant uptake was lower than expected. This led to non-Muslim South Asian men lacking representation in this population, potentially unbalancing the viewpoints of the data and discussion. Lastly, participants of younger ages were missing from this research.

This study has demonstrated that there is both a need but also suggested some mechanisms by which social innovators can improve the physical and mental health of women with vision loss from ethnic minorities through PA. Using a Socio-Ecological Model framework social innovators can provide holistic and cultural equitable PA programs targeted at disabled, ethnic and gender minorities specifically. Social innovators should work with local government and sporting facilities to design accessible (via public transport) opportunities which allow these minority populations to engage in PA whilst upholding their personal values and cultural integrity.

## Data Availability

The dataset analysed during the current study is available from the corresponding author on reasonable request.
